# Strong electric field tuning of magnetism in self-biased multiferroic structures

**DOI:** 10.1038/s41598-020-78104-9

**Published:** 2020-12-03

**Authors:** Xu Li, Christopher S. Lynch

**Affiliations:** 1 Department of Mechanical and Aerospace Engineering, University of California, Los Angeles, CA 90095 USA; 2 Bourns College of Engineering, University of California, Riverside, Riverside, CA 92521 USA

**Keywords:** Nanoscience and technology, Nanoscale devices, Magnetic devices

## Abstract

A new type of multiferroic heterostructure has been proposed in this work with strong electric field tuning of magnetism. It is composed of a self-biased magnetic layered structure with perpendicular magnetic anisotropy (PMA) and one piezoelectric substrate. Two configurations were investigated by a modeling approach, Ni/Ni/Ni/PMN-PT with Cu as spacer and Terfenol-D/CoFeB/Ni/PMN-PT. Magnetic multilayers at their resonance exhibit multiple absorption peaks from acoustic and optical modes of spin interaction between adjacent magnetic layers. A piezoelectric substrate transfers electric field induced strain to adjacent magnetic layer and thus shifts resonance frequencies of the multiferroic structure by tuning magnetic effective fields through magnetoelastic coupling. It has been demonstrated computationally that the resonance frequencies for the simulated structures could be up to 76 GHz under zero magnetic bias field. A larger tunability (> 100%) is achieved with applied electric field to the PMN-PT [011] substrate. Resonance mode selectivity is present in the configuration Terfenol-D/CoFeB/Ni/PMN-PT wherein one desired mode exhibits a much higher tunability compared to other modes. This enables the total mode number to be tuned by merging or diverging different modes under E-field.

## Introduction

With an increasing need on high selectivity, broadband tunability, low insertion loss and compact configuration, multiferroic structures have recently drawn a lot of attention due to strong magnetoelectric effect for future filter applications. Magnetic material’s response to an external field is proportional to its susceptibility, which has a non-linear dependence on frequency. The magnetic response reaches maximum when spin precession frequency matches excitation field frequency. This phenomenon is called ferromagnetic resonance (FMR). The system has the highest energy absorption rate at resonance. Based on this characteristic, magnetic materials can be used to design a bandstop filter with only certain frequency range of electromagnetic signals being absorbed. Spin precession frequency depends on the effective field of magnetic layers. In other words, FMR frequency can be shifted by applied magnetic field. Electric field induced strain in ferroelectric layer modulates the effective magnetic field in adjacent magnetic layers by magnetoelastic coupling and thus tunes the center frequency of multiferroic structures. This characteristic can be used to design a voltage tuning filters, tunable phase shifters, tunable inductors, etc.

Until now, considerable efforts have been directed at investigating single layer magnetic film on top of a piezoelectric substrate, such as FeGaB/PZN-PT^1^, BaFe_12_O_19_/Ba_0.5_Sr_0.5_TiO_3_^2^, BaFe_12_O_19_/PMN-PT^3^, FeGaB/PZN-PT^4^, YIG/PMN-PT^5^. These experiments observed mainly one significant absorption peak on magnetic materials which can be tuned by applied electric field. In telecommunication system, multiband filter with tunable band number and tunable band central frequency is of great interest. A good example is multiple bands in mobile devices support roaming between different regions where different standards are used for mobile telephone services. Particularly, satellite communication systems work in Ku-band or Ka-band which require much higher working central frequency, 10–40 GHz, for direct broadcast satellite services or military purpose.

In this work, a multimode tunable heterostructures with magnetic layered configuration is proposed which can achieve three resonance modes compared to maximum two modes in existing work^1–6^. Each magnetic layer in the proposed structure is designed in a way to display different perpendicular PMA. Surface anisotropy induced PMA gives a high central frequency 2–12 GHz even under zero magnetic bias field, significantly higher than 900 MHz reported in Zavislyak et al.^5^, and 0.2 GHz observed in Vukadinovic et al*.*^8^. Tatarenko et al*.* reported a resonance frequency at 6 GHz but a bias magnetic field 1700Oe was required to obtain such high frequency^3^. There is also a large tunability 7.8–10.7 GHz in the present work while in Das et al. a maximum 40 MHz shift can be achieved by a 9 V bias voltage^2^, in Tatarenko et al. a maximum 120 MHz tunability was observed^3^, and another research reported a tunability of 20 MHz^5^. It has been demonstrated that the effective field gradient in layered magnetic structures is essential to achieve large tunability of the heterostructures in this work. This magnetic layered structure is placed on the top of a PMN-PT, which is used to tune the structures via magnetoelastic coupling. Both resonance frequencies and mode number can be tuned by electric field. This proposed multimode tunable structure has a broad potential application in the communication system which is able to meet various design requirements with desired filter band number, large frequency tunability (> 100%) and tuned frequency band selectivity.

## Methods

A frequency domain model which couples micromagnetics and linear piezoelectricity was used in this work. Micromagnetics is described by the linearized Landau–Lifshitz–Gilbert (LLG) equation in frequency domain^7^

1 $$ i\omega \delta \underline{m} - (\gamma \mu_{0} \underline{{H_{eq} }} + i\alpha \omega \underline{{m_{eq} }} ) \times \delta \underline{m} + \gamma \mu_{0} \underline{{m_{eq} }} \times \underline{{H_{T} }} \left( {M_{s} \delta \underline{m} } \right) = - \gamma \mu_{0} \underline{{m_{eq} }} \times \delta \underline{h} $$

where $\mu_{0}$ is the vacuum permeability, *γ* is the gyromagnetic ratio, *α* is the Gilbert damping constant, $M_{s}$ is saturation magnetization, $H_{eq}$ is total effective field at equilibrium state, which includes an external bias field $H_{ext}$, PMA $H_{PMA}$, magnetostatic field (demagnetization field) $H_{demag}$, exchange field $H_{ex}$ , magnetoelastic field $H_{me}$. $\delta \underline{h}$ is external excitation field, and $\delta \underline{m}$ is magnetization deviation away from its equilibrium state under excitation field. $\underline{{H_{T} }}$ is effective field change due to magnetization deviation $\delta \underline{m}$. PMA energy density is expressed as $- \left( {\frac{{K_{s} }}{d} + K_{v} } \right)M_{{_{3} }}^{2}$ where $K_{s}$ is the surface anisotropy constant, $d$ is the film thickness, $K_{v}$ is the PMA coefficient for Terfenol-D at 2 nm, and $M_{{_{3} }}^{{}}$ is the magnetization component along the out-of-plane direction. Expressions for other energy terms can be found in Li, et al.^17^.

Bulk magnetic materials without PMA generally exhibit in-plane multidomain states resulting from the competition between exchange energy and magnetostatic energy^15^. Therefore, an external bias field along the in-plane direction is required in bulk magnetic materials in order to obtain a quasi-single-domain magnetic state, which is critical to achieving a significant absorption peak in FMR application^5,15^. However, in this work ultra-thin magnetic films with PMA were utilized. PMA arises from the missing half-plane of atoms in ultra-thin magnetic films^13,17^, which favors an easy axis along the out-of-plane direction. Thin magnetic films with PMA do not require external bias magnetic field to achieve a quasi-single-domain state, since PMA overcomes the demagnetization field and forces magnetizations to stabilize along one direction, which is the out-of-plane easy axis direction. This characteristic was referred to as “self-bias” in Li et al*.*^6^.

Equation (1) is further rearranged by introducing three math tensors, $\underline{\underline{{D_{1} }}}$, $\underline{\underline{{D_{2} }}}$ and $\underline{\underline{{D_{3} }}}$^7^

2 $$ \delta \underline{m} = - \left( {i\omega \underline{\underline{I}} - \underline{\underline{{D_{2} }}} + \underline{\underline{{D_{1} }}} \underline{\underline{{D_{3} }}} } \right)^{ - 1} \underline{\underline{{D_{1} }}} \underline{\delta h} = \underline{\underline{\chi }} \left( \omega \right)\underline{\delta h} $$

where three math tensors are defined as $\underline{\underline{{D_{1} }}} \delta \underline{m} = \mu_{0} \left| \gamma \right|\underline{{m_{eq} }} \times \delta \underline{m}$, $\underline{\underline{{D_{2} }}} \delta \underline{m} = \left( {\left| \gamma \right|\mu_{0} \underline{{H_{eq} }} + i\alpha \omega \underline{{m_{eq} }} } \right) \times \delta \underline{m}$ and $\underline{\underline{{D_{3} }}} \delta \underline{m} = \underline{{H_{T} \left( {\delta \underline{m} } \right)}}$, respectively. $\underline{\underline{\chi }} \left( \omega \right)$ is a 2nd order frequency-dependent dynamic susceptibility tensor that indicates the amplitude of the magnetic response to external excitation field. This term reflects the power absorption by the structure.

In order to capture spin interaction between adjacent magnetic layers, Hoffman boundary condition is applied at interface, which states as^8^

3 $$ \underline{{m_{i} }} \times \left[ {2A_{i} \frac{{\partial \underline{{m_{i} }} }}{{\partial \underline{{n_{i} }} }} + \nabla_{mi} E_{s,i} - J_{ij} \underline{{m_{j} }} } \right] = 0 $$

where $\underline{{m_{i} }}$ is magnetization vector in i_th_ layer (i = 1,2,3), $A_{i}$ is exchange constant in i_th_ layer, $E_{s,i}$ is surface anisotropy for the same layer, J_ij_ is interface exchange constant between i_th_ and j_th_ adjacent magnetic layer(j = 1,2,3, j ≠ i). The effective field matrix at equilibrium state for magnetic layered structure in Cartesian coordinate is given in Eq. (4)

4 $$ H_{eq} = \left\lfloor {\begin{array}{*{20}c} {\left[ {H_{eq1} } \right]_{3 \times 3} } & {\left[ { - H_{e1 + } } \right]_{3 \times 3} } & 0 \\ {\left[ { - H_{e2 - } } \right]_{3 \times 3} } & {\left[ {H_{eq2} } \right]_{3 \times 3} } & {\left[ { - H_{e2 + } } \right]_{3 \times 3} } \\ 0 & {\left[ { - H_{e3 - } } \right]_{3 \times 3} } & {\left[ {H_{eq3} } \right]_{3 \times 3} } \\ \end{array} } \right\rfloor $$

This is 3 × 3 matrix with 9 cells. Each cell is also a 3 × 3 matrix which represents equilibrium magnetic field along Cartesian coordinates. The diagonal cells are the equilibrium magnetic fields in each layer, while off-diagonal terms are the effective fields from spin interaction between adjacent layers. This effective field tensor is then plugged into Eq. (1) to obtain magnetospectrum of magnetic multilayer structure.

Resonance frequencies of magnetic layered structure are tuned by electric field induced piezo-strain through magnetoelastic interaction. Behavior of piezoelectric substrate follows piezoelectricity^9^.

5a $$ \underline{\underline{\sigma }} = \underline{\underline{{\underline{\underline{{C^{E} }}} }}} \underline{\underline{\varepsilon }}^{el} - \underline{\underline{{\underline{e} }}} \underline{E} $$

5b $$ \underline{D} = \underline{\underline{{\underline{{e^{T} }} }}} \underline{\underline{\varepsilon }}^{el} + \underline{\underline{\xi }} \underline{E} $$

where $\underline{D}$ is the electric displacement, $\underline{E}$ is the electric field. $\underline{\underline{{\underline{\underline{{C^{E} }}} }}}$ is the elastic stiffness tensor measured under constant E. $\underline{\underline{\xi }}$ is the permittivity tensor. $\underline{\underline{{\underline{e} }}}$ is the piezoelectric coupling tensor. Electric field induced strain is transferred to adjacent magnetic layer which alters its effective field through magnetoelastic coupling. Since ferromagnetic resonance (FMR) frequency and higher order ones are all dependent on the effective fields of magnetic films, the resonance frequency of structures can thus be tuned due to modified magnetic field through magnetoelastic coupling. Note that in this work, macro-spin model is used for each magnetic layer with all the spins perpendicular to device surface in their equilibrium state. Critical parameters used in this simulation is given in Table 1. Since no specific interlayer exchange constant for Ni, CoFeB and Terfenol-D layer was available, a reasonable value range estimated from previous work^10–12^ is chosen for the simulation in this work, 0.5–3 × 10^–3^ J/m^2^, given that the coupling strength can be easily tailored by the spacer properties.

**Table 1:** Critical parameters for E-field tuning heterostructure design.

Properties	Value
Interlayer exchange constant^10–12^	0.5–3 × 10^–3^ [J/m^2^]
Surface anisotropy constant for Ni, K_s_^13^	3.2 × 10^–4^ [J/m^2^]
Surface anisotropy constant for CoFeB, K_s_^14^	1.3 × 10^–3^ [J/m^2^]
PMA constant for Terfenol-D, K_v_^14^	3.4 × 10^–5^ [J/m^3^]
Damping coefficient for Ni , $\alpha$^15^	0.038
Damping coefficient for CoFeB , $\alpha$^14^	0.01
Damping coefficient for Ni , $\alpha$^14^	0.06
Bulk magneto-elastic coupling constant for Ni, B_1_^15^	7 × 10^6^ [J/m^3^]
Bulk magneto-elastic coupling constant for CoFeB, B_1_^14^	− 6.2 × 10^6^ [J/m^3^]
Bulk magneto-elastic coupling constant for Terfenol-D, B_1_^14^	− 1.4 × 10^8^[J/m^3^]

## Results and discussion

**Figure 1:**
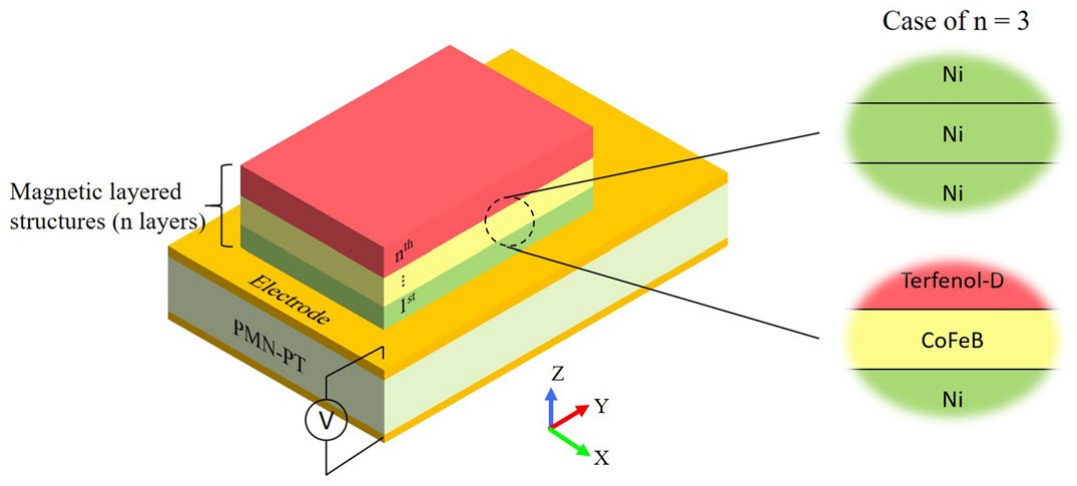
Simulation configuration for E-field tuning multiferroic heterostructure, composed of a magnetic layered structure and a piezoelectric substrate. Two magnetic layered structure configurations are proposed, Ni(2 nm)/Ni(4 nm)/Ni(6 nm)/PMN-PT and Terfenol-D(2 nm)/CoFeB(1.8 nm)/Ni(6 nm)/PMN-PT.

Proposed multiferroic heterostructure configuration is given in Fig. 1. All three magnetic layers exhibit PMA with out-of-plane equilibrium magnetization state. PMA arises from surface anisotropy of magnetic materials, which is inversely proportional to film thickness. Multiple works reported PMA in Ni film when thickness was below 7 nm^13,17^ and in CoFeB/Terfenol-D films when thickness is below 2 nm^14^. Two magnetic layered structure are thus proposed, Ni(2 nm)/Ni(4 nm)/Ni(6 nm)/PMN-PT[011] with Cu Spacer and Terfenol-D(2 nm)/CoFeB(1.8 nm)/Ni(6 nm)/PMN-PT[011]. Different thickness for each magnetic layer was chosen, which gives effective field discrepancy among different layers. In Ni/Ni/Ni/PMN-PT structure, 2 nm, 4 nm and 6 nm were selected for the simulation. However, one can choose any thickness below 7 nm for Ni film to tailor the magnetic resonance behavior as needed. Same in Terfenol-D/CoFeB/Ni/PMN-PT structure, different sets of thickness (≤ 2 nm for CoFeB/Terfenol-D films, < 7 nm for Ni film) can be selected to meet customized application requirements. In simulation, PMN-PT have Au plate electrodes on both sides. One side is sourced with DC electric signal while the other side is grounded. When this device is subject to an external electromagnetic signal, spins in three layers start to oscillate around their equilibrium state which come to resonance at certain frequencies. Therefore, proposed structure only absorbs the electromagnetic signal at resonance modes and allows the signal with other frequencies passing through. This characteristic can be used to design a multiband tunable band-stop filter, wherein both band frequency range and band number can be tuned by DC voltage signal applied to the PMN-PT substrate.

**Figure 2:**
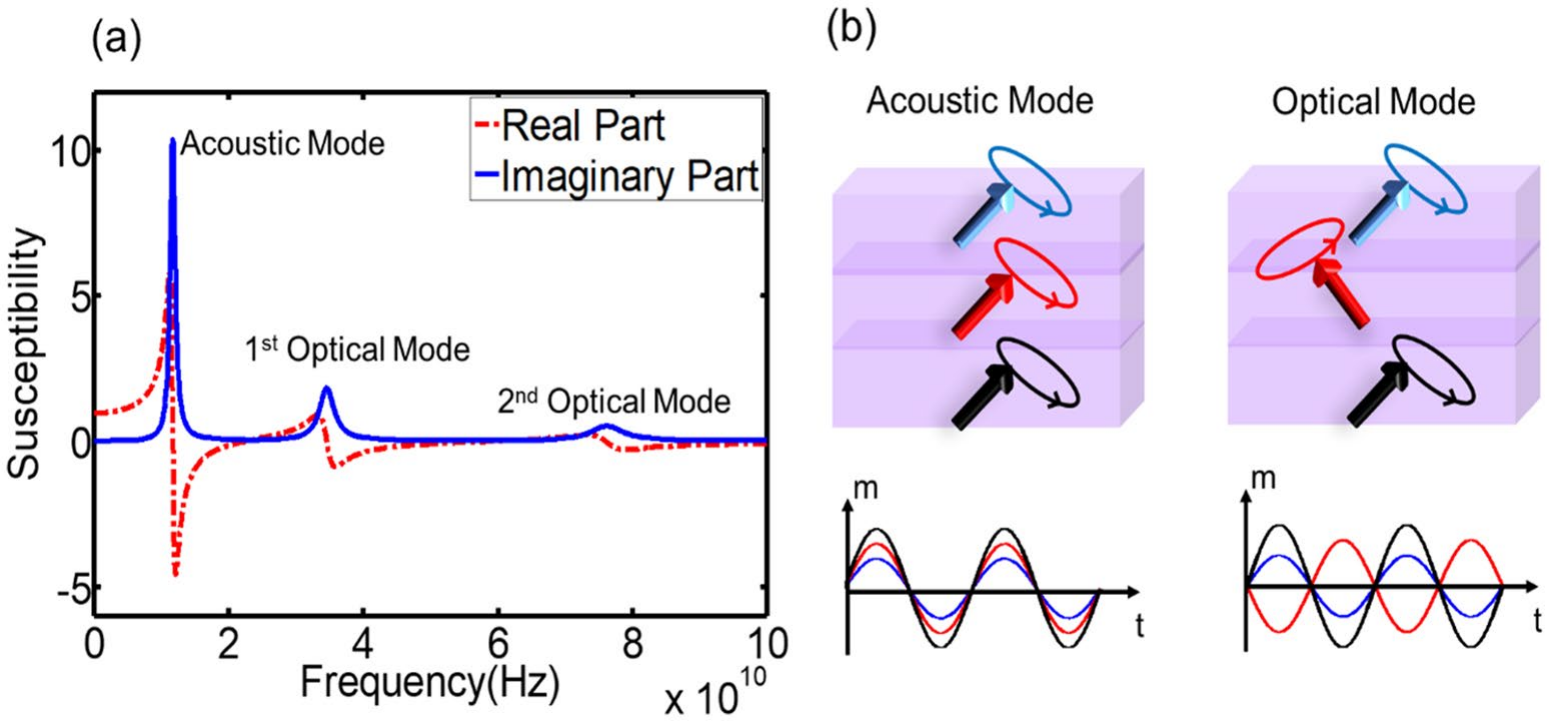
(**a**) Magnetospectrum for Ni/Ni/Ni/PMN-PT[011] multiferroic heterostructure based on simulation results. One acoustic mode and two optical modes are presented. The resonance frequencies are 11.66 GHz, 34.55Ghz, and 76.20 GHz, respectively. (**b**) Illustration of different resonance modes. Acoustic mode, which is also referred as FMR mode or fundamental mode. All the spins oscillate in the same phase. Optical mode, wherein at least one layer rotates out-of-phase with others.

Figure 2a shows the magnetospectrum of configuration Ni/Ni/Ni/PMN-PT [011] multiferroic heterostructure under zero magnetic bias field. Three resonance modes are observed, which are 11.66 GHz, 34.55 GHz, and 76.20 GHz, respectively. The first mode, 11.66 GHz, is usually referred as fundamental FMR mode or acoustic mode. Under this mode, all spins oscillate in the same phase, as illustrated in Fig. 2b. Therefore, the total response of the system to the external electromagnetic signal reaches maximum. Higher order resonance modes (optical modes), 34.55 GHz, and 76.20 GHz, also present in the system resulting from spin interaction between adjacent magnetic layers. Under optical modes, spins oscillate with different phases in each layer, and thus reduces the total response of the system. Figure 2b shows one scenario of optical modes with middle layer’s spins rotating out of phase with the other two layers. The real part of susceptibility represents the energy exchange between multiferroic heterostructures and electromagnetic field with spin precession in phase with external excitation field. The imaginary part of susceptibility is proportional to the power dissipation. Energy irreversible absorption rate, or power dissipation, in magnetic film is expressed as^16^ $P_{abs} = - \frac{{\omega \mu_{0} }}{2}\iiint\limits_{v} {{\text{Im}} \left[ {H_{res}^{*} \chi H_{res} } \right]}dV$, where H_res_ is the resonance magnetic field. Therefore, a larger imaginary part of susceptibility indicates a higher energy absorption rate. When frequency is equal to zero, absolute susceptibility value gives static response of the system. This figure demonstrates that even with zero magnetic bias field, the proposed structure still displays high resonance frequencies (> 10 GHz) by taking advantage of self-biased PMA. Multiple absorption modes are also obtained resulting from spin interaction between adjacent magnetic layers.

**Figure 3:**
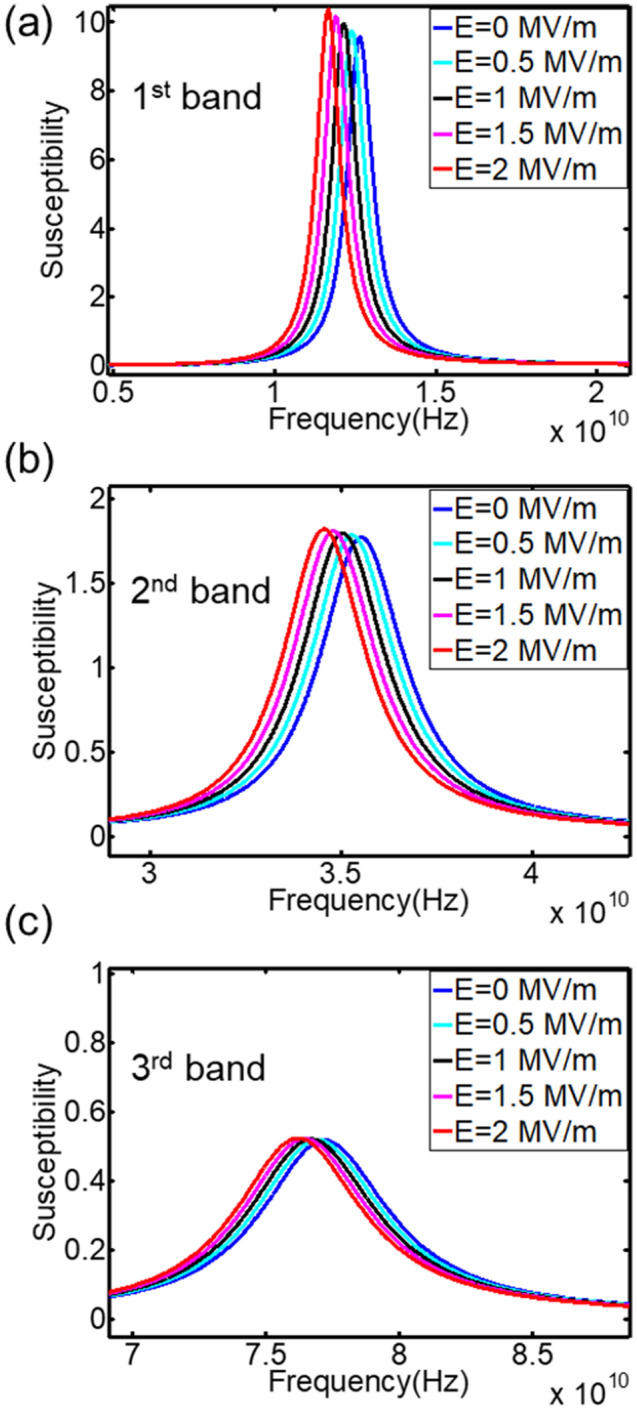
Computational magnetospectrum for Ni/Ni/Ni/PMN-PT[011] with Cu as spacer under ramping electric fields. (**a**) First mode tunability. (**b**) Second mode tunability. (**c**) Third mode tunability.

In order to better understand different resonance modes, phase information for each magnetic layer under all three modes are given in Table 2. Under acoustic mode, all three layers have almost the same phase, which is around − 86°. Here minus (−) sign means magnetization response lags the excitation field by 90°. Correspondingly, plus (+) sign means magnetization response advances the excitation field by 90°. Under 1st optical mode with resonance frequency 34.55 GHz, the 2 nm Ni layer and the 4 nm Ni layer are oscillating in phase, while they both have around 180° phase difference from the 6 nm layer. For the 2nd optical mode with resonance frequency 76.20 GHz, none of magnetic layers is in phase, as shown in Table 2. This phase difference cancels out the total response of magnetic films, which results in low energy absorption rate from electromagnetic signal. Figure 3 shows the E-field tunability of this configuration. A ramping voltage is applied to the PMN-PT [110], which generates electric field varying from 0 to 2 MV/m. Figure 3a–c are the magnetospectrum shifts for three modes. Since all three magnetic layers are Ni film, there is a same amount of effective magnetic field shift in each layer under piezo-strain. Therefore, three resonance frequencies are decreasing by 0.98 GHz simultaneously with increasing electric fields.

**Table 2:** Spin precession phases for each magnetic layer under three resonance modes.

Mode	Resonance frequency (GHz)	All layers (º)	Ni(6 nm) (º)	Ni(4 nm) (º)	Ni(2 nm) (º)
Acoustic	11.66	− 85.77	− 87.07	− 83.87	− 77.16
Optical	34.55	− 88.90	104.12	− 88.50	− 82.15
Optical	76.20	− 93.63	− 117.90	110.33	− 88.84

**Figure 4:**
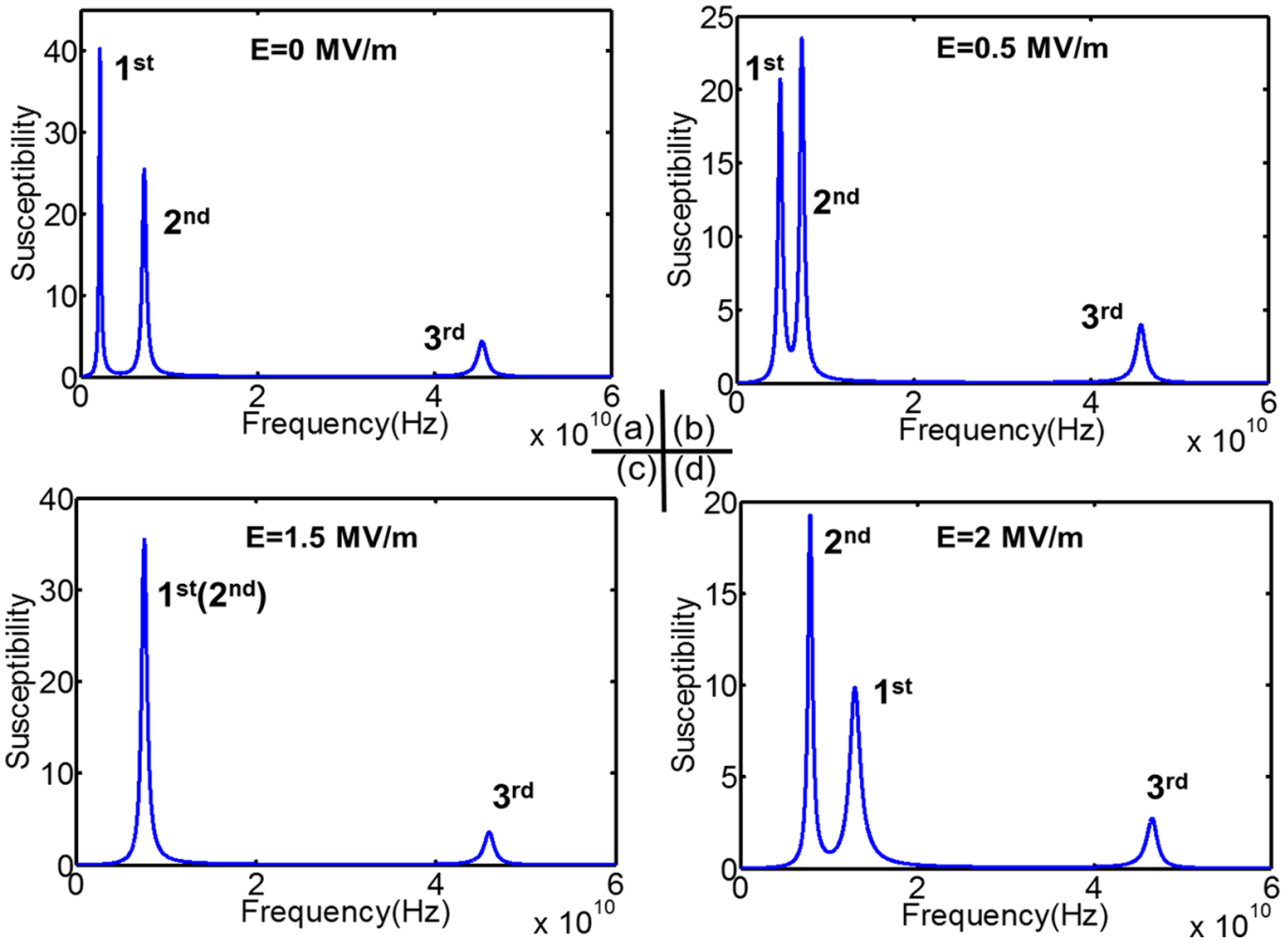
Computational Zero-H-field magnetospectrum for Terfenol-D/CoFeB/Ni/PMN-PT[110] multiferroic structures under ramping electric fields. (**a**) E = 0 MV/m. Three resonance modes are observed, 2.21 GHz, 7.22 GHz and 45.39 GHz, respectively. (**b**) E = 0.5 MV/m. The 1st mode is closer to the 2nd mode. (**c**) E = 1.5 MV/m, the 1st mode is merged with the 2nd mode. Only two modes are observed at this point. (**d**) E = 2MV/m. The original 1st mode travels further towards the 3rd mode. Structures again display three modes.

**Figure 5:**
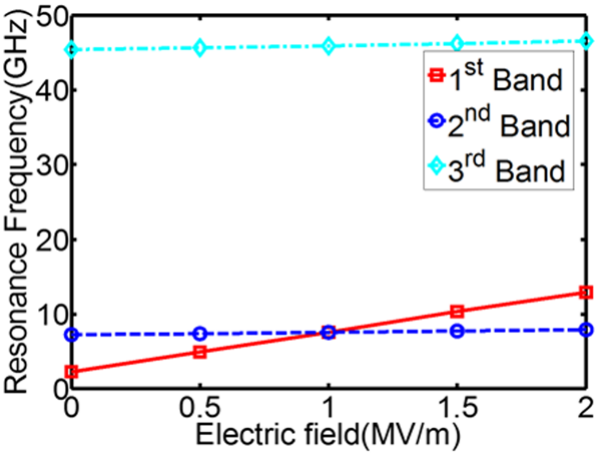
Frequency tunability of Terfenol-D/CoFeB/Ni/PMN-PT[110] multiferroic structures under a ramping electric field, which shows selectivity over 1st frequency band or mode.

Another configuration of multiferroic tunable structures was proposed in order to achieve tuned mode selectivity and larger frequency tunability, that is Ni(6 nm)/CoFeB(1 nm)/Terfenol-D(2 nm)/PMN-PT[110] heterostructure. Saturation magnetostriction coefficient for Terfenol-D is 1200 ppm, which is about 45 times higher than Ni, − 27 ppm. CoFeB is also a positive magnetostrictive material with $\lambda_{s} = 50\;{\text{ppm}}$. Since both Terfenol-D and CoFeB are positive magnetostriction materials, the magnetoelastic field induced by tensile strain along z axis reinforces spins stabilized along that direction. Ni is a negative magnetostriction material. 2 MV/m electric field generates 1200 ppm strain in z direction, which has been shown to be not enough to overcome the energy barrier between out-of-plane and in-plane direction^17^. Therefore, the equilibrium state for the Ni film is still along the z-direction. This characteristic enhances the stiffness field discrepancy in different magnetic layers when it is subjected to electric fields. A ramping voltage is applied to the PMN-PT[110], which generates electric field varying from 0 to 2 MV/m. Figure 4a is the magnetospectrum under zero E-field and zero H-field wherein three modes are presented, 2.21 GHz, 7.22 GHz and 45.39 GHz. Upon applying electric field, the 1st mode shifts to the right with ramping electric field, as indicated by Fig. 4b,c. When electric field increases to 1.5 MV/m, the 1st mode is merged with the 2nd mode. Therefore, only two modes are observed at this point. When electric field continues to increase, the original 1st mode shifts further to the right. The system again displays three modes (Fig. 4d). Figure 5 shows the frequency tunability for all three modes during this process. The 2nd resonance frequency only shifts 0.69 GHz ($\frac{\Delta f}{f} = 9.6\%$), and 3rd resonance frequency shifts 1.14 GHz ($\frac{\Delta f}{f} = 2.51\%$). They are barely tuned compared to the 1st mode, which shifts 10.7 GHz ($\frac{\Delta f}{{f_{{}} }} = 484.2\%$ ). The simulation results indicate that Terfenol-D/CoFeB/Ni/PMN-PT[110] multiferroic structure can achieve tuning only on one resonance mode while other modes remain untuned. This feature further enables one to tune the total number of resonance modes by merging or diverging individual mode.

**Figure 6:**
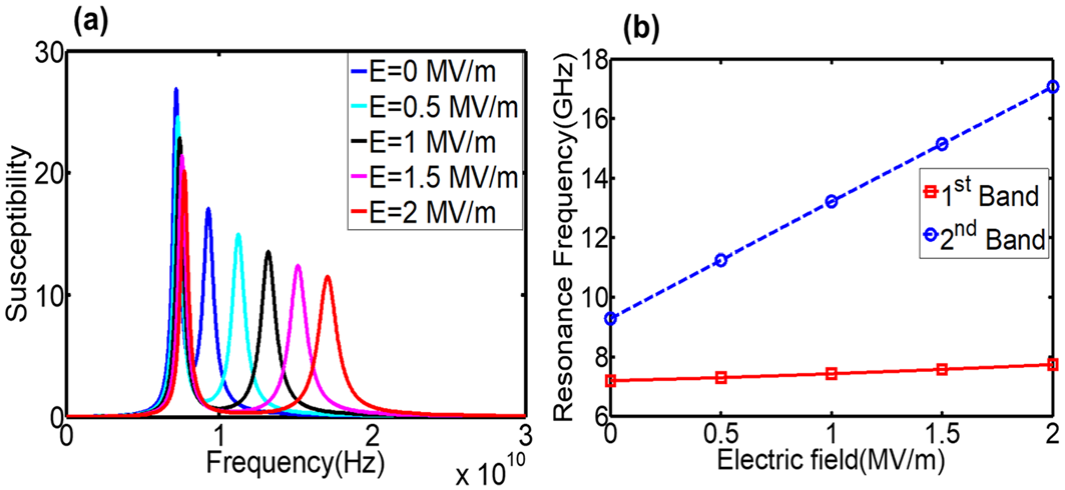
Voltage tunability of Terfenol-D/CoFeB/Ni/PMN-PT multiferroic structures with zero-H-field. Interlayer exchange constant is 3e−3 J/m^2^ between Terfenol-D and CoFeB, and 0.5e−3 J/m^2^ for CoFeB/Ni interface. (**a**) Magnetospectrum under ramping electric fields. (**b**) Resonance frequency shifts in terms of electric fields. A large tunability up to 7.78 GHz ($\frac{\Delta f}{{f_{{}} }} = 83.74\%$) can be obtained for the 2nd band/mode.

Tuned resonance modes can be selected by tailoring the interlayer exchange constant between magnetic layers. This coupling strength can be altered by spacer properties and the separation distance of two layers. Interlayer exchange energy is expressed as $E = - J_{ij} \underline{{M_{i} }} \cdot \underline{{M_{j} }}$^8^, where M_i_ is magnetization in i_th_ layer. J_ij_ is interlayer exchange coupling constant. Maximum J_ij_ can be determined approximately from the equation $J_{ij} = m\frac{{J^{\prime}S^{2} }}{{a^{2} }}$^10^ or $J_{ij} = \frac{2A}{{M_{i} M_{j} b}}$^11^, where S is the spin number, a is the lattice constant, m = 8 for fcc lattices, $J^{\prime}$ is the exchange integral across the interface, A is the average value of exchange constants for two magnetic layers, and b is the separation of the layers. Both expressions indicate that the interlayer exchange constant can be altered by the separation distance of two layers, i.e. spacer thickness. To selectively tune the 2nd mode instead of the 1st mode, a stronger coupling between Terfenol-D layer and CoFeB layer, J = 3e−3 J/m^2^, is used in the new design. Corresponding E-field tuning magnetospectrum is given in Fig. 6a. Two modes are displayed under zero E-field and H-field, 7.19 GHz and 9.29 GHz. Under ramping electric fields, the 1st mode almost has no shift. However, a large tunability up to 7.78 GHz is obtained in the 2nd mode ($\frac{\Delta f}{{f_{{}} }} = 83.74\%$). It is because spins in the CoFeB and Terfenol-D layers always precess together to yield one absorption peak (1st mode) due to stronger interlayer coupling strength. The 1st mode is the acoustic mode, where all spins in three magnetic films rotate together. Since Ni film has negative magnetostriction, it cancels out total stiffness field shift of CoFeB and Terfenol-D layers when it's subject to ramping electric fields. While under the 2nd mode, Ni is out-of-phase by 180° relative to the other two layers. Due to opposite polarity of magnetostriction, the stiffness field shifts of all three layers are added up for the system in response to electric fields. Therefore, a large tunability is selected to be only displayed in the second mode, as shown in Fig. 6b. This simulation result demonstrates that interlayer exchange coupling can selectively tune one resonance mode while other modes remain untuned.

## Conclusion

An E-field tuning multiferroic heterostructure was proposed in this work, which is consisted of a self-biased magnetic layered structure with PMA and a piezoelectric substrate. It utilizes small oscillation behavior of spins at resonance when it’s subject to external electromagnetic signals. Two examples were investigated using a frequency domain simulation, Ni(2 nm)/Ni(4 nm)/Ni(6 nm)/PMN-PT[011] with Cu spacer and Terfenol-D(2 nm)/CoFeB(1.8 nm)/Ni(6 nm)/PMN-PT[011]. The proposed structure configurations exhibit up to three absorption peaks resulting from the spin interaction between magnetic layers. High resonance frequencies (> 10 GHz) are displayed in the structures under zero H-field from self-biased PMA of magnetic layers. With ramping electric fields, all three resonance modes are tuned simultaneously in the configuration Ni/Ni/Ni/PMN-PT. For the configuration Terfenol-D/CoFeB/Ni/PMN-PT, only one mode is shifted under electric fields while the others remain almost untuned. This further enables the tunability of total number of resonance modes via merging or diverging individual resonance frequency. It has also been demonstrated that the proposed structure has selectivity over the mode to be tuned by altering interlayer exchange coupling strength. Though this work only presents two examples of the proposed multiferroic heterostructure, it provides lots of design freedom regarding magnetic layer number, magnetic material selection and interlayer exchange coupling strength to produce desired functions. This multiferroic heterostructure opens a broad design space for future multimode tunable devices.

## Data Availability

All data generated or analyzed during this study are included in this published article.
